# COVID-19 transmission sources, management, and scientific research in Turkey

**DOI:** 10.3906/sag-2009-159

**Published:** 2021-04-30

**Authors:** Fatih KAHRAMAN, Adem ÖZKARA

**Affiliations:** 1 Department of Cardiology, Kütahya Evliya Çelebi Research and Training Hospital, Kütahya Turkey; 2 Department of Family Medicine, University of Health Sciences, Ankara City Hospital, Ankara Turkey

## To the Editor,

Severe acute respiratory syndrome coronavirus 2 (SARS-CoV-2) was seen first in Wuhan, China and spread very quickly all over the world despite very strict precautions taken worldwideWHO (2020). Novel coronavirus—China [online]. Website https:// www.who.int/csr/don/12-january-2020-novel-coronavirus-china/en/ [accessed 00 Month Year].. The World Health Organisation (WHO) declared the disease a pandemic on March 11, 2020. Turkey reported its first case of the coronavirus on the same day and had started taking precautions both in the country and along the borders months earlier. Over time, many case reports and remarkable articles relating to the disease progression, virus genomics, and treatment options were published in high-quality journals. Simultaneously, country-wide data registries and research activities began in Turkey. Recently, a correspondence sent from Turkey offering a critique of some important points concerning the disease, including transmission routes to Turkey (mainly Iran and Saudi Arabia), lack of transparency at the Turkish Ministry of Health (MoH), and, in particular, scientific research restrictions on COVID-19 was published in Lancet, one of the most prestigious medical journals [1]. We read the correspondence of Hasan Bayram et al. with great interest. It reported important information concerning COVID-19 transmission routes to Turkey, a lack of transparency by the Turkish MoH, and purported restrictions on scientific research on COVID-19.

We would like to address some concerns mentioned by Bayram et al. For instance, the authors claim that COVID-19 reached Turkey mainly through Iran and Saudi Arabia; however, research indicates many different origins for the disease in Turkey. The SARS-CoV-2 genome was phylogenetically analyzed in Turkish laboratories and in laboratories around the world, and the results were documented in the global initiative on sharing all influenza data (GISAID) platformGlobal Influenza Surveillance and Response System (2020). Genomic epidemiology of hCoV-19 [online]. Website https://www.gisaid.org/epifluapplications/ next-hcov-19-app/ [accessed 15 August 2020].. Karacan et al. analyzed the viral isolates of three COVID-19–positive patients and found that all three isolates carried the D614G variant in the S gene, indicating that they are all in the G clade, which was mostly detected in European countries [2]. In addition, Adebali et al. performed a phylogenetic analysis of the first thirty SARS-CoV-2 genomes isolated and sequenced in Turkey [3]. Their analysis suggested multiple independent international introductions of the virus to the country. They clustered the genomes based on their clade distribution in the phylogenetic tree and found that most samples from Turkey belong to cluster four, which is also prevalent in Iran, Denmark, and France. They found a connection to Saudi Arabia in only two cities in Turkey, while the Europe-based introductions were identified in the genomes isolated from İstanbul, the epicenter of Turkey’s COVID-19 outbreak. In addition, according to Turkish Ministry of Culture and Tourism data on border introduction counts between January and March, Iran and Saudi Arabia accounted for only 6.72% of all visitors (5.19% from Iran and 1.53% from Saudi Arabia). During the same interval, entry from Europe constituted 38.52% of all visitors (24.88% from OECD countries and 13.79% from non-OECD countries)Republic of Turkey Ministry of Culture and Tourism (2020). Turkey borders entrance and exit reports (in Turkish) [online]. Website https://yigm.ktb.gov.tr/TR-256539/2020.html [accessed 00 August 2020].. The first COVID-19–positive cases were seen within the same day in Iran and Europe, nearly 20 days before Turkey’s first documented case. Furthermore, Turkey closed its border with Iran on February 23 after Iran’s first cases of COVID-19 were confirmed on February 19; however, flights to and from Italy, one of the European countries most affected by COVID-19, were not cancelled until February 29. Additionally, Turkish visitors returning from pilgrimages in Saudi Arabia were checked with thermal cameras before boarding and were quarantined in select isolated hostels for 14 days. Considering all this well-documented information, attributing virus introduction to only one or two sources seems illogical.

The authors’ next point, that there is a lack of transparency at the Turkish MoH, has already been addressed by the organization in its daily briefings on the MoH website and on social mediaRepublic of Turkey Ministry of Health (2020). Current Status in Turkey [online]. Website https://covid19.saglik.gov.tr/?lang=en-US [accessed 17 August 2020].. The authors’ most controversial subject is the alleged restrictions on COVID-19 research. The rumours about research restrictions are simply not true. In fact, the timely responses of the Turkish MoH to research applications encouraged researchers to work faster and more efficiently. According to data from the official MoH website, nearly 5000 research applications regarding COVID-19 were submitted online, and the MoH approved more than 96% of them within five days, allowing researchers to proceed with their proposed studiesRepublic of Turkey Ministry of Health (2020). Official announcements about scientific research studies on COVID-19 (in Turkish) [online]. Website https://bilimselarastirma.saglik.gov.tr/_layouts/15/BilimselYayin_Membership/login.aspx?ReturnUrl=%2f_layouts%2f15%2fAuthenticate.aspx%3fSource%3d%252F&Source=%2F [accessed 16 August 2020].. Local institutes and councils also supported many trials concerning COVID-19 [2,3].

Finally, in addition to this objective data, Turkey’s official agencies, healthcare workers, and community have come together to quell the COVID-19 outbreak as successfully as any other country. Personal protective equipment (PPE) was produced in collaboration with formal and private agencies and distributed all around the country. Moreover, Turkey has supported many countries, including the United States and the United Kingdom, with PPE. Two large emergency hospitals were built in İstanbul and began providing services in only 45 days. In addition, mechanical ventilators were produced with 100% local resources for the first time and delivered to local hospitals. With mass production, Turkey was able to manufacture enough ventilators to export to other countries. As a result of these proactive and effective steps, Turkey has had lower mortality rates than many developed countries (Table).

**Table:** Fatality and mortality rates calculated according to current population of countries

Countries	Fatality rate	Mortality rate
Turkey	0.024	0.07
United States of America	0.031	0.50
United Kingdom	0.131	0.60
Belgium	0.129	0.86
France	0.152	0.47
Spain	0.083	0.61
Italy	0.139	0.59

1 Worlddometer (2020). Countries in the World by population [online]. Website https://www.worldometers.info/world-population/populationby-country [accessed 16 August 2020]. 2 WHO (2020). COVID-19 Dashboard (death counts) [online]. Website https://covid19.who.int/ [accessed 16 August 2020].
